# Comparison of Epirubicin-Iodized Oil Suspension and Emulsion for Transcatheter Arterial Chemoembolization in VX2 Tumor

**DOI:** 10.1100/2012/961986

**Published:** 2012-03-12

**Authors:** Tatsuo Ueda, Satoru Murata, Takahiko Mine, Shiro Onozawa, Munehiko Onda, Zenya Naito, Yasuo Amano, Shinichiro Kumita

**Affiliations:** ^1^Department of Radiology, Nippon Medical School, 1-1-5 Sendagi, Bunkyo-ku, Tokyo 113-8603, Japan; ^2^Department of Integrative Oncological Pathology, Nippon Medical School, Bunkyo-ku, Tokyo 113-8603, Japan

## Abstract

To compare the antitumor efficacy and safety of transcatheter arterial chemoembolization (TACE) by epirubicin suspension (epirubicin suspension: epirubicin-iodized oil mixture without solution) to that by epirubicin emulsion (epirubicin emulsion: epirubicin-iodized oil mixture with solution), the efficacy of treatment by administration of either an epirubicin suspension or emulsion was examined in an animal model. Changes in plasma epirubicin concentration were compared over 24 h immediately after treatment, and enhanced ultrasonographic and histopathological analysis subsequently conducted 7 days after treatment to determine the growth ratio and proportion of viable tumor cells. The growth ratio and proportion of viable tumor cells were found to be significantly lower in the suspension group than in the emulsion group while the plasma epirubicin concentration was found to be significantly higher in the suspension group than in the emulsion group. These results indicate that administration of an epirubicin suspension is a superior form of TACE compared to that of administration of an epirubicin emulsion.

## 1. Introduction

The incidence of hepatocellular carcinoma (HCC), the fourth most common cause of cancer-related death worldwide, is currently increasing [1]. Fortunately, in developed countries, HCC is diagnosed in 30 to 40% of all patients at the early stages, when it is amenable to treatment, such as surgical therapy and percutaneous treatment [2]. However, no systemic therapy has yet been developed that improves the survival of patients with advanced HCC [3, 4]. One effective treatment may be administration of sorafenib (Nexavar; Bayer HealthCare Pharmaceuticals-Onyx Pharmaceuticals, Leverkusen, Germany), a molecular target-based drug that a recent study found could improve prognosis modestly [5].

Whereas 80% of the blood to healthy liver tissue is supplied by the portal vein, 99% of the blood to hepatic tumors is delivered by the hepatic artery. 

Based on this observation, transcatheter arterial chemoembolization (TACE), a more recent interventional treatment for HCC that is appropriate for patients for whom surgical or percutaneous ablative treatment is contraindicated, currently plays a major role in treating advanced HCC. Support for its use was provided by the results of 2 randomized controlled trials and a meta-analysis, which indicated its survival benefits [6–8]. The protocol for TACE is injection of both iodized oil and anticancer agents into the hepatic artery, followed by administration of embolic agents [9, 10]. Several anticancer agents are used in TACE, including mitomycin, doxorubicin, and cisplatin. Among these agents, epirubicin, the anticancer agent examined in this study, is one of the most commonly used in treating advanced HCC worldwide [11]. Although doxorubicin-eluting beads (DC Beads; Biocompatibles, UK Ltd.) have recently been used as part of a novel drug-delivery embolization system, their superiority to other agents has not yet been proven [12], nor has their use been approved in several countries, including Japan.

As maximizing antitumor efficacy requires transfer of an adequate drug concentration to the target cancer cells for retention in the lesion for a sufficient period [13], drug-iodized oil mixtures that provide sustained release are preferred agents in TACE. Among the types of mixtures that can be used in TACE, water-in-oil (water droplets dispersed in oil) emulsions have been found to exert a stronger embolic effect in HCC treatment compared to oil-in-water (oil droplets dispersed in water) emulsions [14], as well as a greater capacity for sustained release [14, 15]. Although several studies have confirmed that suspensions are more effective than emulsions in providing sustained drug release [15–17], no study to date has compared the antitumor efficacy of suspensions and emulsions of an anticancer drug in experimentally induced liver tumors.

To fill this research gap, this study compared the antitumor efficacy and safety of TACE performed by administration of an epirubicin-iodized oil suspension to that of TACE performed by administration of an epirubicin-iodized oil emulsion in the treatment of experimentally induced liver tumors in an animal model.

## 2. Materials and Methods

### 2.1. Animals

All experiments were performed using 10 female Japanese white rabbits (2.9–3.5 kg; mean, 3.06 kg; SLC, Tokyo, Japan) according to a protocol that had been approved by the Animal Experiment Ethics Committee of the university at which the study was conducted. Prior to study initiation, the VX2 carcinoma strain, a known model of hypervascular tumor, had been maintained by successive transplantation into the hindlimb of a carrier rabbit. 

Experimentation began 3 weeks after 0.1 mL of minced VX2 carcinoma had been implanted into the subcapsular parenchyma of the left medial liver lobe of the 10 rabbits through a midline abdominal incision. In all experiments, the rabbits were anesthetized by intravenous injection of thiopental sodium (40 mg/(kg·h)^−1^; Ravonal, Tanabe-Mitsubishi, Osaka, Japan).

### 2.2. Experimental Groups

After all 10 rabbits had been inoculated with VX2 carcinoma cells, 5 were randomly assigned to the emulsion group (weight range 2.9–3.2 kg; mean, 3.01 kg) and 5 to the suspension group (weight range 2.9–3.5 kg; mean, 3.08 kg). The emulsion group was administered 0.5 mg/kg of epirubicin (Lipiodol Ultra-Fluide; Laboratoire Guerbet, Aulnay-Sous-Bois, France) in 0.1 mL/kg of iodized oil with 0.1 mL/kg saline solution while the suspension group was administered 0.5 mg/kg of epirubicin in 0.1 mL/kg of iodized oil without saline solution. To prepare the emulsion, 10 mg epirubicin was dissolved in 2 mL of saline solution, of which 2 mL (5 mg/mL) had been mixed with 2 mL of iodized oil by use of a pumping method consisting of 20 pushes and pulls through a stopcock between 2 disposable syringes (1 push and pull per 2 s) immediately before infusion [18]. To prepare the suspension, 10 mg of epirubicin was mixed directly with 2 mL of iodized oil by manual stirring, followed by use of the same pumping method used to prepare the emulsion immediately before infusion.

### 2.3. TACE Procedure

**Figure 1:**
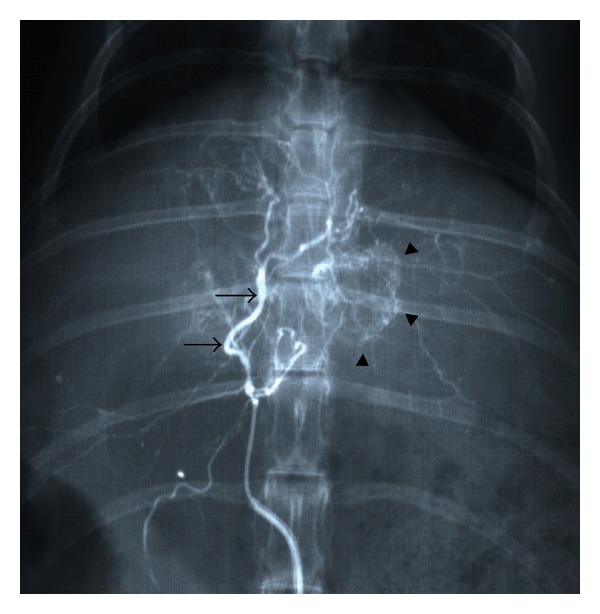
TACE procedure. The therapeutic agent was administered via a 2.3-Fr microcatheter inserted into the middle hepatic artery (arrows), which can be seen feeding the tumor (arrowheads).

Three weeks after implantation of the VX2 carcinoma, TACE was performed by experienced interventional radiologists (T. Ueda, S. Murata, T. Mine, and S. Onozawa) under fluoroscopic guidance. After general anesthesia had been induced, the right femoral artery was exposed by an incision for insertion of a 4-Fr sheath (Medikit Co., Tokyo, Japan). Celiac angiography was performed with a 4-Fr Cobra-type catheter (Medikit Co.) to reveal the hepatic arterial anatomy and identify the artery feeding the tumor. The middle hepatic artery, which exclusively supplied blood to the tumor in all the rabbits, was selectively catheterized using a 2.3-Fr microcatheter (Transit; Cordis, Miami, FL). After the catheter had been advanced to a suitable position in the artery, the emulsion or suspension was carefully injected to avoid effluxion of the embolic material (Figure 1). The catheter was then removed, and the femoral artery ligated.

### 2.4. Tumor Size Analysis

**Figure 2:**
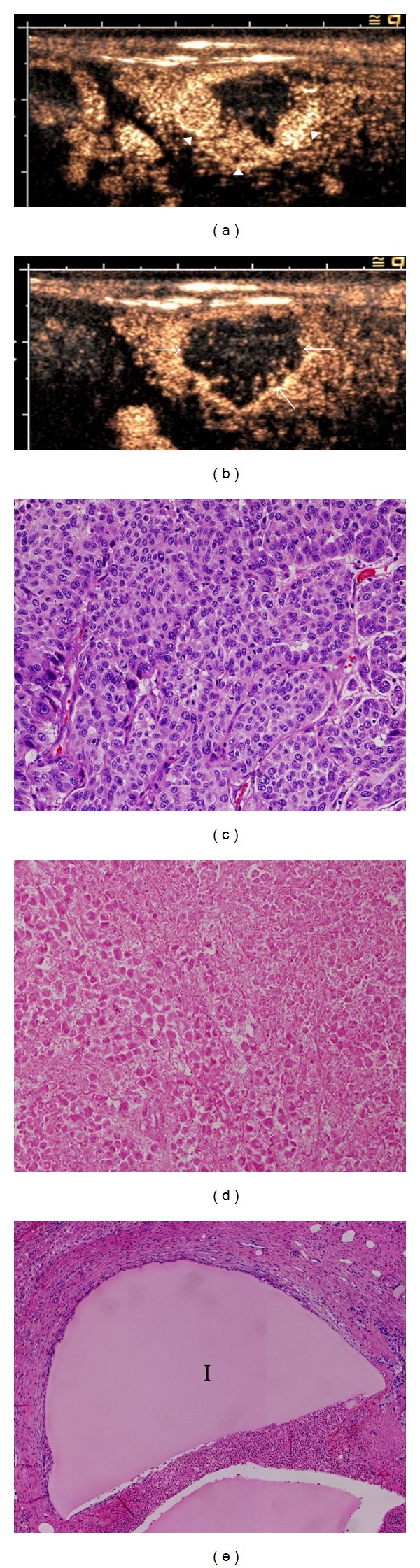
Sonazoid-enhanced ultrasonographic and histopathological examination. (a) Immediately after Sonazoid administration, the tumor shows a strong contrast effect, depending on the vascular distribution, particularly in the marginal area (arrowheads). (b) While the tumor lacks a contrast effect 10 min after administration of Sonazoid (the Kupffer phase), the edge can be clearly visualized (arrows). (c) Cellular colonies with adequately stained nuclei and cytoplasm can be observed in the viable (c) but not the necrotic portion (d). A deposit of iodized oil (*I*) remains in the destroyed vascular structure (e). (c–e): hematoxylin-eosin staining, 20x original magnification.

Tumor size was measured by performing abdominal ultrasonography (Acuson Sequoia 512; Siemens Medical Systems, Erlangen, Germany) with an electronic linear probe (15L8W) immediately before and 7 days after TACE with the animals in the supine position. Enhanced ultrasonography was enabled by a single injection of 0.015 mL/kg of Sonazoid (Daiichi-Sankyo, Tokyo, Japan) in the auricular vein. Using the images obtained by Kupffer-phase scanning (20 frames/s; 10–13 MHz; cadence contrast pulse sequence mode) at 10-min intervals, tumor location and size in the sagittal section were measured 3 times by experienced radiologists (T.U. and M.T.) and the average size was calculated by consensus (Figures 2(a) and 2(b)). The area (*A*) of the tumor, which was considered to assume an elliptic shape, was calculated using the formula *A* = *L* × *S*/4 × 3.14, where *L* and *S* are the longest and shortest diameters of the tumor, respectively. The average of 3 consecutive measurements of the diameter was utilized for the calculation. The growth ratio was calculated by comparing the tumor area obtained before (*A*_0_) and 7 days after (*A*_7_) treatment using the formula ((*A*_7_/*A*_0_) − 1) × 100.

At 7 days after TACE, the rabbits were sacrificed by overdose of thiopental sodium for histological investigation. After extirpation, the tumors were sliced sagittally and measured macroscopically. The macroscopic measurements were evaluated statistically and compared to the ultrasonographic measurements to determine the accuracy of the latter.

### 2.5. Histopathological Examination

After extirpation, the rabbit livers were fixed in a 20% formaldehyde solution. The tumor-containing portion was sliced at 5 mm intervals in the sagittal plane to correspond to the plane of the ultrasound imaging. The resulting 4 *μ*m sections were embedded in paraffin and stained with hematoxylin and eosin for histological examination, with the largest and second largest tumor sections also utilized for pathological investigation. Cellular colonies for which adequately stained nuclei and cytoplasm that could be observed under microscopic examination were classified as viable, while colonies for which inadequate nuclear staining or lacking nuclei or cytoplasm that could be observed were classified as necrotic (Figures 2(c)–2(e)). Experienced pathologists (M. Onda and Z. Naito) evaluated the histological changes and extent of necrosis in the tumors. Antitumor effects were quantified by estimating the viable proportion of the entire tumor, with the overall proportion of the viable tumor calculated on the basis of the mean percentage in each slice.

### 2.6. Pharmacokinetics and Toxicity

Blood samples were collected from the auricular vein at 0, 0.5, 1, 3, 6, and 24 h immediately after administration. The samples were centrifuged and the plasma was utilized for measuring the epirubicin concentration, an indicator of the extent of sustained action. The extent of hepatic and hematological toxicity experienced by the 2 groups was evaluated by drawing peripheral blood from the auricular vein before and at 1, 3, 5, and 7 days after TACE for measurement of plasma aspartate aminotransferase (AST), alanine aminotransferase (ALT), and total bilirubin (T-Bil) levels.

### 2.7. Statistical Analysis

The correlation between ultrasonographic and direct measurements of tumor size was evaluated by calculating Pearson's correlation. The antitumor efficacy (i.e., impact on tumor growth ratio and viable proportion) of the 2 forms of treatment was compared by performing Mann-Whitney *U* testing. Plasma epirubicin concentration and biochemical levels were compared by performing unpaired *t* testing. SPSS version 14 (SPSS Japan, Tokyo, Japan) was used to perform all statistical analyses and all *P* values less than 0.05 were considered statistically significant. All data are presented as the mean ± standard deviation.

## 3. Results

TACE was performed successfully for all rabbits, as evidenced by the 0% mortality rate over the 7-day treatment period. Blood samples were obtained over the study period and microscopic sections immediately after the treatment period from all rabbits.

### 3.1. Change in Tumor Size

**Figure 3:**
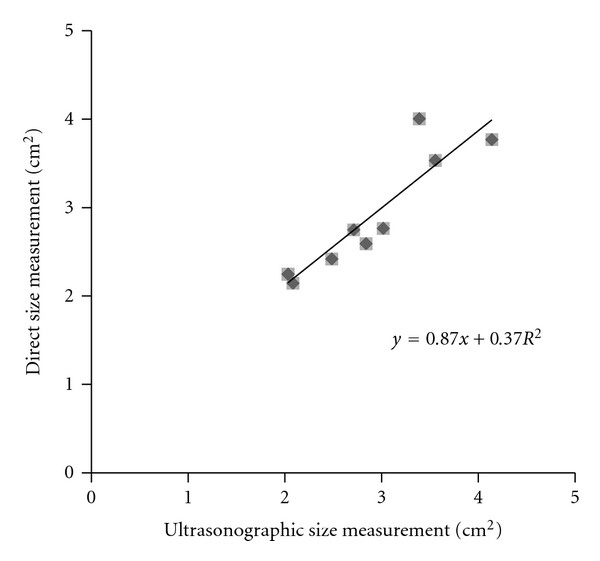
Correlation between ultrasonographic and direct (macroscopic) measurements of tumor size. Pearson's correlation coefficient (*R* ^2^) between the ultrasonographic and the direct measurements was found to be 0.84 (*n* = 10, *P* < 0.001).

**Figure 4:**
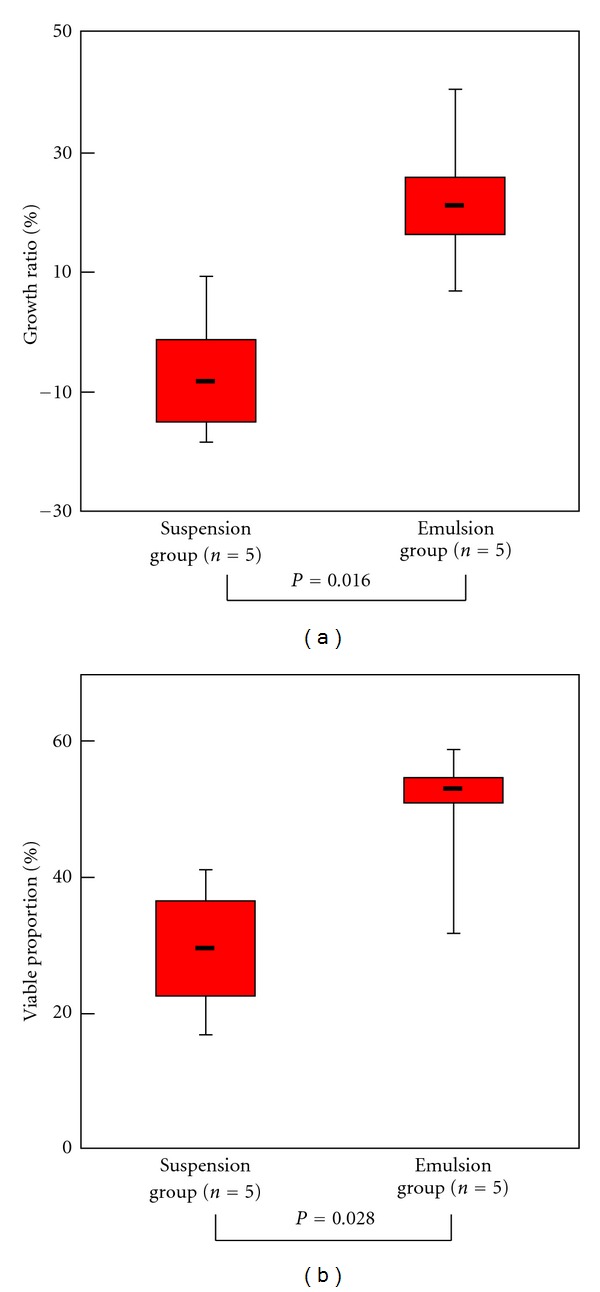
Growth ratio and viable proportion. (a) Graph showing the macroscopically determined growth ratio of liver tumors 7 days after TACE. The tumors in the suspension group showed minimal growth or slight shrinkage over the experimental period, while those in the emulsion group exhibited a mild increase in volume. The growth ratio of the tumors in the suspension group was found to be significantly smaller than that in the emulsion group (*P* = 0.016, Mann-Whitney *U* test). (b) Graph showing the viable proportion of liver tumors at 7 days after TACE. The percentage of residual viable tumor cells ranged from 16.6% to 41.1% in the suspension group and from 31.6% to 58.7% in the emulsion group. The proportion of viable tumor cells was found to be significantly smaller in the suspension group than in the emulsion group (*P* = 0.028, Mann-Whitney *U* test).

The difference between the mean tumor sizes at baseline (*A*_0_  ± SD) of the emulsion and suspensions groups, which were calculated as 2.35 ± 0.75 cm^2^ and 3.04 ± 0.44 cm^2^, respectively, was not found to be statistically significant (*P* > 0.05, Mann-Whitney *U* test). Statistical comparison of the results of ultrasonographic examination 7 days after treatment (*A*_7_), which revealed that the tumor area ranged from 2.03 to 4.14 cm^2^ (2.83 ± 0.7 cm^2^), and macroscopic analysis, which revealed that the tumor size ranged from 2.14 to 4.00 cm^2^ (2.85 ± 0.7 cm^2^), indicated a significant correlation between the ultrasonographic and direct measurements of tumor size (*R* ^2^ = 0.84, *P* < 0.001; Figure 3). During the experimental period, the tumors in the suspension group were observed to exhibit minimal growth or slight shrinkage (Figure 4(a)) and a growth ratio ranging from −18.4 to 9.4% (−7.3 ± 11.3%). In contrast, the tumors in the emulsion group were found to display a significantly smaller growth ratio (*P* = 0.016, Mann-Whitney *U* test) and an increase in volume ranging from 6.6% to 40.4% (21.4 ± 12.6%).

### 3.2. Viable Proportion

On microscopic examination, both viable and necrotic tumor cells were observed in all specimens. The proportion of viable tumor cells depended on vessel damage, with strong vessel damage leading to a relatively smaller viable proportion. Statistical comparison of the viable proportion of the tumors in the suspension group, which ranged from 16.6 to 41.1% (28.0 ± 10.4%), and the emulsion group, which ranged from 31.6 to 58.7% (49.6 ± 10.5%), indicated that the proportion of viable tumor cells was significantly smaller in the suspension group than in the emulsion group (*P* = 0.028, Mann-Whitney *U* test; Figure 4(b)).

### 3.3. Pharmacokinetics and Toxicity

**Figure 5:**
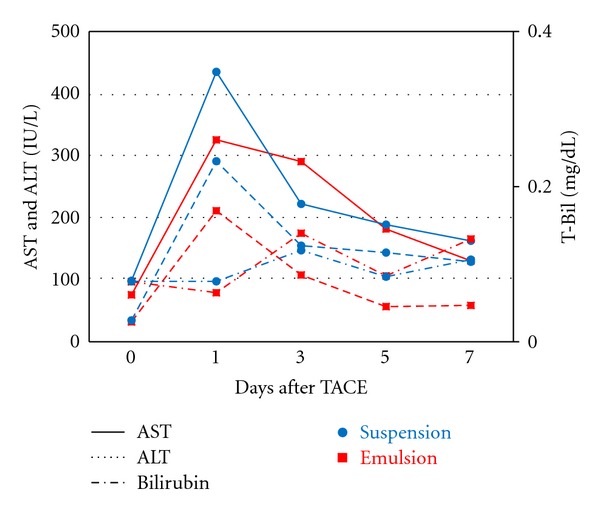
Levels of toxicity. AST and ALT levels increased for several days after TACE, and did not recover to baseline levels during the experimental period. Although the levels of the suspension group increased to a slightly greater extent compared to those of the emulsion group, the differences between the levels of the 2 groups did not reach a level of statistical significance. No significant changes were observed in the T-Bil level of either group.

Table 1 shows the sequential changes observed in plasma epirubicin concentration. At 1 h after administration, the concentration of the suspension group was found to be significantly higher than that of the emulsion group (*P* = 0.02, unpaired *t* test). However, the difference in concentration between the 2 groups did not reach a level of statistical significance at 0.5, 3, 6, and 24 h after administration. As can be observed in Figure 5, which shows the plasma AST and ALT levels before and after TACE, AST and ALT levels were transiently but significantly elevated at 24 h after TACE, but decreased to approximately normal levels at 7 days after TACE. No significant differences were found in the AST or ALT levels between the 2 groups over the study period nor were any significant changes in plasma T-Bil levels observed in either group.

## 4. Discussion

As an embolic material, iodized oil has several characteristics, including a high level of viscosity, that allows it to destroy peribiliary plexus or peripheral arterioles [19]. Moreover, as iodized oil is distributed according to the vascularity of organs or tumors, it can be selectively deposited for an extended period in areas that have become necrotic due to its injection [20].

Histopathologically, simultaneous administration of anticancer drugs and iodized oil emulsions has been found to provide better therapeutic effects than administration of iodized oil alone [21, 22]. Specifically, the results of randomized trials of transcatheter arterial-infusion therapy with epirubicin with and without iodized oil, which found that administration of an epirubicin-iodized oil emulsion was more effective than that of epirubicin alone (response rate, 42% and 12%, resp.) [23], suggest that administration of anticancer-drug-iodized-oil emulsions exerts more potent antitumor effects than administration of iodized oil or drugs alone.

In the treatment of portal vein ischemia, which results in injury to the portal vein endothelium and thrombus formation, anticancer drugs contained in iodized oil have been found to exert antitumor effects. Specifically, iodized oil has been observed to traverse the sinusoids from the portal venules into the hepatic vein, where oil droplets are immediately washed into the systemic circulation. The time required for clearance of iodized oil from the liver and recovery of the microcirculation depends largely on the patency of the hepatic artery. The iodized oil activates Kupffer cells, which capture and phagocytose the oil in hepatic sinusoids and likely exert a synergistic tumoricidal effect toward hepatic tumors [24]. As such, TACE-induced anticancer effects and damage to tumor and/or peritumoral vessels are closely related [25].

In this study, evaluation of the combined antitumor effects of epirubicin and iodized oil in a group administered the treatment in suspension form and a group administered the treatment in an emulsion group revealed a significant correlation between the ultrasonographic and macroscopic measurements of the tumors (*R* ^2^ = 0.84, *P* < 0.001), indicating that tumor size can be accurately measured by Sonazoid-enhanced ultrasonography. The growth ratio in the suspension group was found to be smaller than that in the emulsion group, indicating that providing epirubicin in suspension form yields a much stronger antitumor effect compared to administering it in emulsion form. This indication was supported by the results of histopathological analysis, which revealed that administration in suspension form led to more severe damage to the tumor and/or peritumoral vessels, and thus a much better antitumor effect.

Microscopic analysis of the extent of necrosis of tumor cells caused by ischemia due to vessel damage, with a low proportion of viable tumor cells indicating greater vessel damage [25], revealed that the area of viable tumor cells depends on the extent of vessel damage. Specifically, greater vessel damage, which causes intimal proliferation, thrombi, and degenerative changes, leads to a relatively decreased viable tumor area.

The administration of sustained-release anticancer agents can reduce drug leakage into the peripheral blood, allowing for maintenance of a high local drug concentration in tumors and drug distribution to plasma at a relatively slower rate. Research has demonstrated that the release rate of anticancer agents can be controlled by modifying the form in which they are administered, with the suspension form being found superior to the emulsion form in maintaining sustained agent release [15–17]. In this study, the suspension form (without water) was found to remain within the tumor and plasma for a longer period compared to the emulsion form (with water). As the distribution half-life of epirubicin is extremely brief (approximately 4.67 min) [26], the highest epirubicin concentration in both groups was observed immediately after injection.

While the epirubicin concentration in both groups was observed to decrease rapidly 0.5 h after administration, the concentration in the suspension group was found to be significantly higher than that in the emulsion group at 1 h after administration. This finding indicates that epirubicin remained within the tumors for a longer period and was distributed more slowly to the plasma in the suspension group, suggesting that the suspension form provides a greater extent of sustained release compared to the emulsion form. However, this conclusion has a significant caveat: as a significant difference in plasma concentration was observed at a single isolated point—at 1 h after administration—this observation might be able to be attributed to the very short half-life of epirubicin. Therefore, the next step in this line of research is investigation of the time-concentration curve of epirubicin within tumors for more specific analysis of the antitumor effects of the suspension.

In accordance with previous clinical research, which found that liver enzymes commonly increase transiently following TACE, peaking at 24–36 h before returning to baseline levels at 5–7 days [27–30], the AST and ALT levels of all the rabbits in this study were found to increase transiently before returning to baseline levels over the 7-day experimental period. Moreover, no significant differences in liver enzyme levels were observed between the groups, demonstrating the safety of both the suspension and emulsion forms. However, the increase in liver enzymes was found to be slightly greater in the suspension group, which might have been related to greater damage to the tumors and/or peritumoral vessels in this group. These results suggest that intra-arterial administration of epirubicin in suspension form allows for its accumulation in a liver tumor, and may thus be a useful method for reducing systemic toxicity.

The fact that very little research [18] has been conducted into the use of an epirubicin-iodized oil suspension for treating liver tumors may be attributed to the water solubility of epirubicin, a characteristic that poses difficulty in creating a suspension. Indeed, difficulty in mixing epirubicin with iodized oil was a major limitation of this study, requiring that the suspension be prepared using manual stirring and a pumping method. Therefore, efficient preparation of epirubicin-iodized oil suspensions remains a major concern.

In conclusion, this study found that administration of an epirubicin-iodized oil suspension yields superior anticancer efficacy compared to administration of an epirubicin-iodized oil emulsion while posing a relatively low risk of toxicity in the treatment of hepatic tumors. As the next stage in the research into the best form of HCC treatment, a randomized controlled trial will be conducted to compare the antitumor effects of TACE with an epirubicin-iodized oil suspension to that of TACE with an epirubicin-iodized oil emulsion.

## Figures and Tables

**Table 1:** Plasma epirubicin concentration.

Time	Suspension form	Emulsion form	*P*
(h)	(ng/mL)	(ng/mL)	
0	205.73 ± 120.14	173.20 ± 110.58	0.69
0.5	3.53 ± 1.25	2.18 ± 1.90	0.26
1	3.55 ± 1.05	1.26 ± 1.22	0.02
3	2.33 ± 0.85	1.38 ± 1.33	0.26
6	1.06 ± 1.93	1.32 ± 1.42	0.81
24	0.98 ± 1.28	0.62 ± 0.85	0.63

Values are expressed as mean ± SD (*n* = 5 each).
